# A Tooth at Birth

**Published:** 1883-06

**Authors:** Fordyce Grinnell

**Affiliations:** Physician to Pine Ridge Agency, Dakota


### A Tooth at Birth.

Editor The Medical News.

Sir: Yesterday I drew a tooth for an infant seventeen days old. The child was a female, one-quarter Sioux. When it came into the world it had this tooth, a lower central incisor, developed as now presented.

The crown and body of the tooth seemed fully developed, and as large as the usual milk tooth. The attachment to the gum, instead of a root, appeared to be a mere fleshy connection, and hence not very solidly implanted. Moreover, it gave the child great uneasiness in nursing. The tongue in moving over the sharp surface presented by the tooth, in every attempt at drawing milk from the breast, became abraded, and finally ulcerated. As soon as the tooth was extracted, the child took the breast with avidity. The pain caused by nursing had undoubtedly interfered with the child getting a sufficient amount of nourishment.

I thought this case sufficiently unique to justify at least a brief reference to it. Yours, etc.,

Fordyce Grinnell.

*Physician to Pine Ridge Agency, Dakota.*

